# Recovery of high specific activity molybdenum-99 from accelerator-induced fission on low-enriched uranium for technetium-99m generators

**DOI:** 10.1038/s41598-021-92704-z

**Published:** 2021-06-24

**Authors:** M. Alex Brown, Nathan Johnson, Artem V. Gelis, Milan Stika, Anna G. Servis, Alex Bakken, Christine Krizmanich, Kristin Shannon, Peter Kozak, Amanda Barnhart, Chad Denbrock, Nicolas Luciani, Terry Grimm, Peter Tkac

**Affiliations:** 1 Chemical and Fuel Cycle Technologies, Argonne National Laboratory, 9700 S Cass Ave, Lemont, IL 60439 USA; 2 Niowave, Inc., 1012 N Walnut St., Lansing, MI 48906 USA; 3 Radiochemistry Department, University of Nevada Las Vegas, Las Vegas, NV 89154 USA

**Keywords:** Drug development, Nuclear chemistry

## Abstract

A new process was developed to recover high specific activity (no carrier added) ^99^Mo from electron-accelerator irradiated U_3_O_8_ or uranyl sulfate targets. The process leverages a novel solvent extraction scheme to recover Mo using di(2-ethylhexyl) phosphoric acid following uranium and transuranics removal with tri-*n*-butyl phosphate. An anion-exchange concentration column step provides a final purification, generating pure ^99^Mo intended for making ^99^Mo/^99m^Tc generators. The process was demonstrated with irradiated uranium targets resulting in more than 95% ^99^Mo recovery and without presence of fission products or actinides in the product.

## Introduction

The non-invasive diagnosis of a number of vital organ diseases and cancers relies on short-lived γ-emitting isotopes^1^. The most commonly utilized isotope, which comprises over 80% of nuclear medicine today, is ^99m^Tc (*t*_½_ = 6.0 h) which is normally derived from its transient equilibrium parent ^99^Mo (*t*_½_ = 66 h). Today, ^99^Mo is produced using dedicated isotope production reactors^2,3^. A recent surge of interest in utilizing the more proliferation-resistant low-enriched uranium (LEU) under the American Medical Isotope Production Act (AMIPA) has presented the technical challenge: can a domestic supplier meet the estimated weekly U.S. demand of 1500 6–day Ci of ^99^Mo without HEU?

As the production technologies of ^99^Mo pivot towards LEU or molybdenum targets, new reaction channels and accelerators are being evaluated. Superconducting electron linear accelerators (LINACs) with high-Z converter targets can generate bremsstrahlung photons and neutron fluxes that are capable of inducing photonuclear reactions and LEU fission^4^. A particular advantage of this utility is that it does not rely on HEU-fueled reactor cores (which are currently slated for LEU conversion) and can operate on an almost continuous basis. After sufficient production intervals, targets can be rotated out and processed while another batch is irradiated. Although ^99^Mo production rates are lower than conventional HEU reactor channels, the continuous operation capability of electron accelerators, combined with lower costs, reduced waste generation, and their proliferation-resistant nature renders this technology competitive.

Regarding the chemical processing and purification of ^99^Mo from irradiated uranium targets in acidic solutions, the benchmark procedure is known as the Cintichem process or modifications thereof with respects to the use of LEU^5, 6^. The process relies on multiple precipitation, filtration, and column purification steps. It is also important to note that the Cintichem process prescribes the addition of stable Mo to carry ^99^Mo on alpha-benzoin oxime, which reduces the specific activity of ^99^Mo (curies of ^99^Mo per gram of total Mo). Although this practice may be sufficient for large batches of ^99^Mo (> 1000 Ci), it could be problematic for smaller-scale processes that pursue the rapid distribution of hundreds of Ci or less. Conventional alumina-based generators are strongly dependent on the specific activity of ^99^Mo and increasing concentrations of Mo will increase the column size and ultimately decrease the concentration of the ^99m^Tc-saline product^7^.

**Figure 1:**
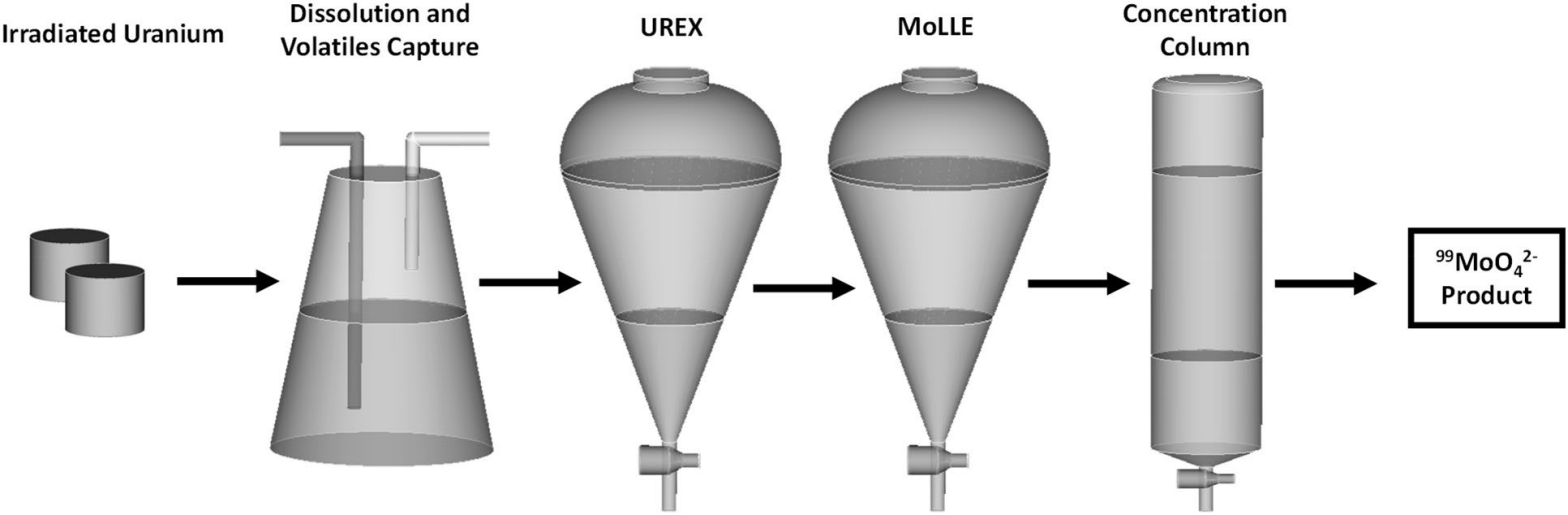
Process overview of the purification steps to produce HSA ^99^Mo from irradiated uranium targets.

Our goal was to develop and experimentally demonstrate a new chemical purification process that could quickly treat irradiated uranium targets for the recovery of high-specific activity ^99^Mo within several hours. To be viable, the procedure must address the following requirements: (1) rapid execution with minimal time-consuming precipitation and filtration steps, (2) no addition of stable carrier elements as to not subvert the high specific activity of valuable fission products, and (3) a method that is mindful of the need to recover LEU target material. To facilitate these requirements, we derived a separation scheme that relies primarily on solvent extraction. This approach results in excellent front-end removal and back-end recovery of uranium in dilute acids (UREX—Uranium Extraction). Second, the solvent extraction of Mo by an organophosphoric or phosphonic acid from mild nitric acids (MoLLE—Molybdenum Liquid–Liquid Extraction) is capable of decontaminating Mo from a mixture of fission products born out of a UREX raffinate^8,9^. Similar process chemistry was recently utilized to selectively remove Mo during the recovery of minor actinides in spent nuclear fuel^10^. The major advantage of this combined approach (UREX + MoLLE) includes the potential to execute these process stages using a continuous flowsheet with equipment such as high-throughput counter-current centrifugal contactors without utilizing time-consuming precipitation and filtration steps. For a final purification, an anion-exchange column yields low volume, high specific activity ^99^MoO_4_^2–^ in a simple alkaline matrix. This platform can also be used to concentrate multiple batches. An overview is illustrated in Fig. 1.

This work summarizes the experimental results of a new chemical process that can rapidly recover microcurie quantities of ^99^Mo from irradiated uranium targets. The discussion will focus on the chemistry surrounding UREX, MoLLE, and the concentration column in order to achieve high purity, high specific activity ^99^Mo. Potential improvements to this process are also discussed.

## Experimental

**Figure 2:**
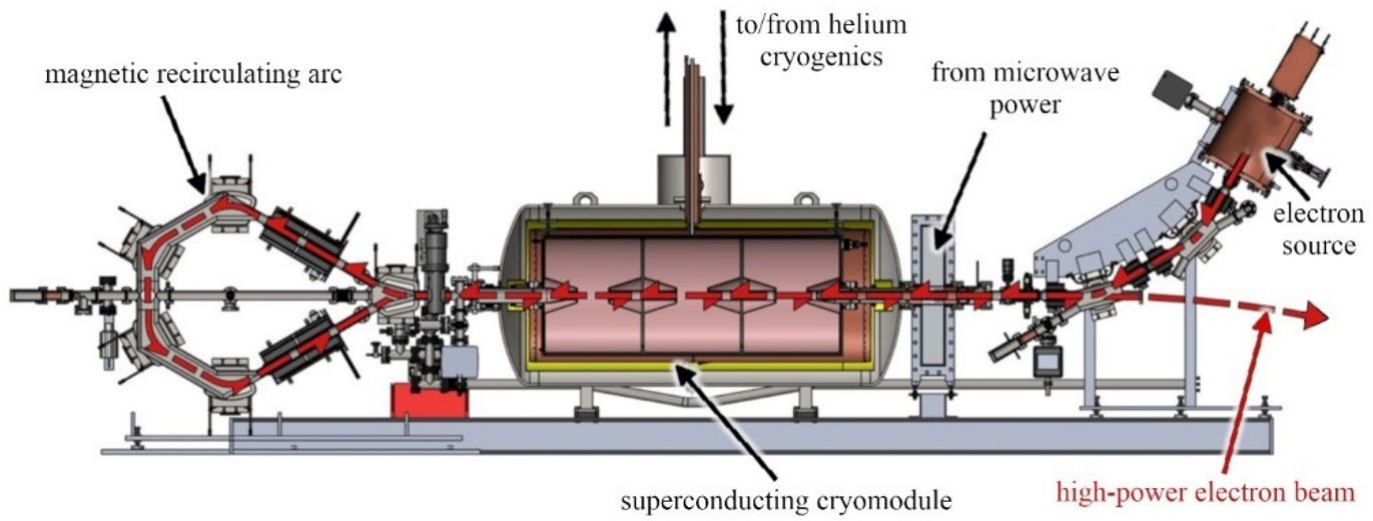
Niowave superconducting electron accelerator, which uses a magnetic recirculation arc to accelerate the electron beam twice in a single superconducting RF structure.

Niowave’s photo-neutron source for isotope production is driven by a superconducting electron accelerator. As shown in Fig. 2, the accelerator is operated in a two-pass configuration to make efficient use of a single superconducting RF structure. The electron beam is available from 5 to 15 MeV, with a capability of up to 40 MeV in full commercial production of ^99^Mo.

Irradiations performed by this system are characterized by the electron beam energy on target (beam power multiplied by irradiation time). For the isotopes described in this paper, 7 kWh of beam was delivered to the isotope target at 9 MeV for 16 h. During production, the energy, power, and the beam positioning is monitored using real-time dosimetry from an ionization chamber and imaging of the Cerenkov light in the cooling water that surrounds the production.

Spectroscopy of pre- and post-irradiation targets as well as radiochemical samples was performed using ORTEC GEM series High Purity Germanium (HPGe) detectors. Each detector was calibrated for energy and efficiency before use with NIST-traceable ^152^Eu volumetric standards acquired from Eckert & Ziegler Isotope Products. Spectrum analysis was performed using PeakEasy 4.86 and decay or transient corrections were applied as necessary^11^.

Two separate uranium targets were irradiated and processed in this study. Following the dissolution or reconstitution of the uranium in 3 M HNO_3_, the irradiated targets were treated under the process flowsheet shown schematically in Fig. 1. Slight iterations were made to the concentrations, volumes, or selected reagents to optimize each stage. First, a natural U_3_O_8_ target (0.71% ^235^U) was obtained from Niowave stocks. The powder was pressed into 12.1 mm × 12.5 mm (diameter × height) and irradiated in Niowave’s Uranium Target Assembly. The target was retrieved, γ-analyzed, and then submitted for processing. The irradiated U_3_O_8_ pellet was dissolved in 16.2 mL of room temperature 6.46 M HNO_3_ for approximately 30 min to yield a feed solution containing 1.3 M uranyl nitrate and 3 M HNO_3_. After the dissolution was complete, the liquor was γ-analyzed, transferred to a 250 mL separation funnel, and emulsified for 1 min with two rounds of 43 mL of 30% (v/v) tri-*n*-butyl phosphate (TBP) in *n*-dodecane to remove the bulk uranium. The raffinate (12.2 mL) was contacted with 13 mL of 0.4 M di(2-ethylhexyl) phosphoric acid (HDEHP) in *n*-dodecane, emulsified for 3 min, and separated after phase disengagement. The loaded HDEHP was transferred to another separation funnel and contacted with two rounds of 13 mL 0.3 M acetohydroxamic acid (AHA) to strip Mo. The Mo-AHA streams were combined and contacted with concentrated NH_4_OH until pH ~ 13.

The second set of targets were comprised of uranyl sulfate solutions. Two irradiations were conducted at Argonne National Laboratory’s Low Energy Accelerator Facility (LEAF) using a ~ 15 kW, 40 MeV beam on a solution of uranyl sulfate (19.75% ^235^U pH 1). Irradiation #1 lasted 6.3 h totaling 54 kW-hr; irradiation #2 lasted 24.3 h totaling 277 kW-h. After a one-week cooling period, an aliquot from each target was added to 5 mL of a depleted uranyl nitrate solution (1.3 M UO_2_^2+^, 3 M HNO_3_) and treated as a feed stock. This feed was contacted with three rounds of 6.7 mL of 30% TBP (v/v) in *n*-dodecane for 45 s to remove the bulk uranium. The raffinate (3.5 mL) was contacted with two rounds of 0.5 mL 0.4 M HDEHP for 45 s. The Mo was stripped from the loaded HDEHP using two contacts of 2.5 mL 0.5 M AHA and adjusted to pH ~ 12 using NH_4_OH.

The concentration column comprised 1.0 g of AG-1X8 (Millipore Sigma) or AG MP-1 (Bio-Rad) that was pre-equilibrated with concentrated NH_4_OH to replace the chloride form. The resin was packed with glass wool in a 1 cm × 10 cm non-jacketed, chromatography column. The column was packed and washed with 1 M NH_4_OH, followed by the feed solution containing ^99^Mo, fission products, and ^239^Np. The wash solutions contained various concentrations of NaOH, HCl, oxalic acid, NaCl, or mixtures thereof described below.

## Results & discussion

**Figure 3:**
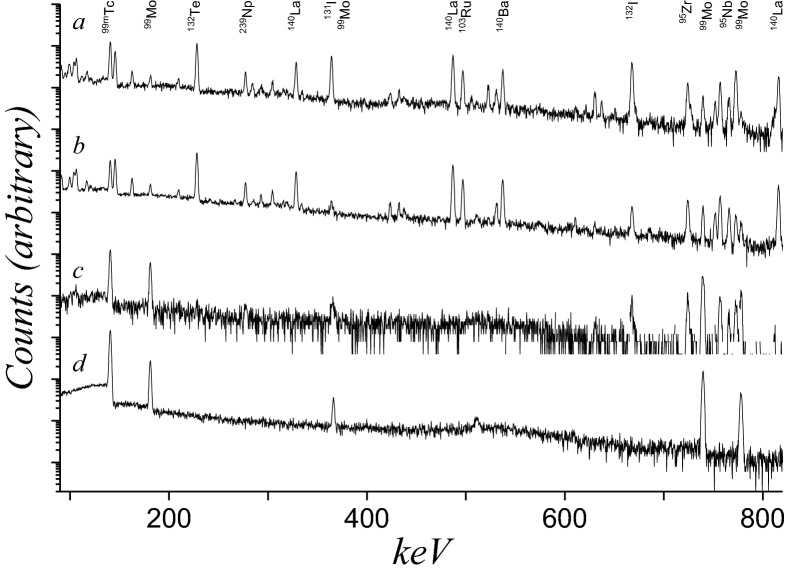
HPGe spectra of the (**a**) UREX feed, (**b**) MoLLE feed, (**c**) concentration column feed, and the (**d**) ^99^Mo product derived from the irradiated uranyl sulfate target (LEU). For clarity only selected isotopes are labelled. Spectra were collected at varying count times.

**Figure 4:**
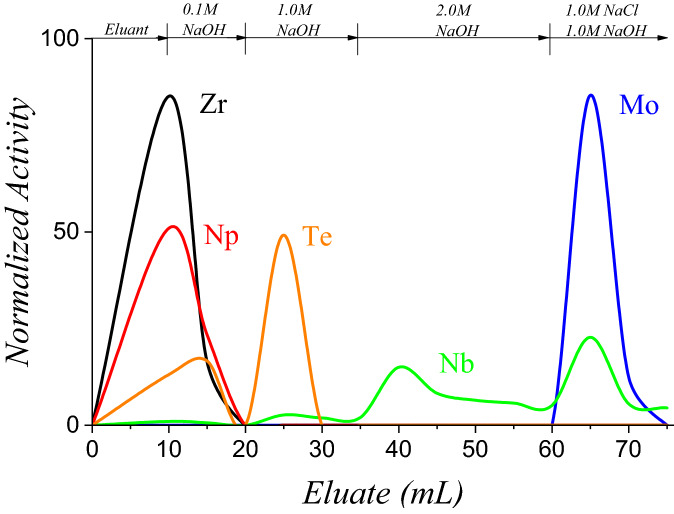
Elution profile of Zr, Np, Te, Nb, and Mo on an anion exchange resin. The feed solution was derived from the irradiated natural U_3_O_8_ target MoLLE raffinate. Column dimensions: 1.0 g AG-MP1 in a 10 cm × 1 cm ID support; flow rate ≈ 2 mL/min.

**Figure 5:**
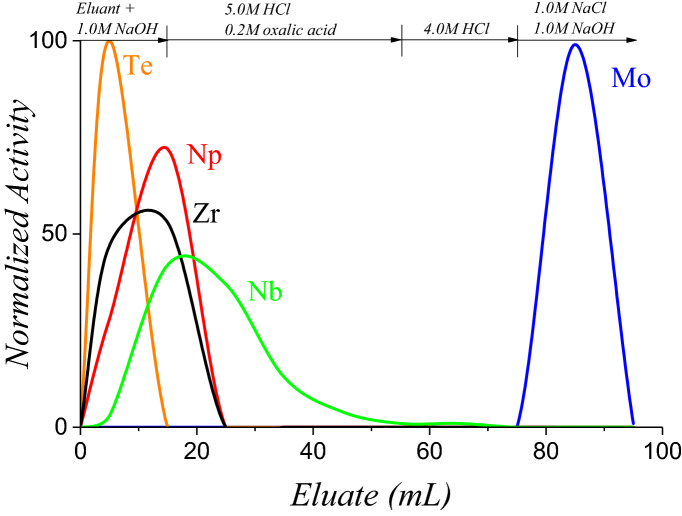
Elution profile of Zr, Np, Te, Nb, and Mo on an anion exchange resin. The feed solution was derived from the irradiated uranyl sulfate target MoLLE raffinate. Column dimensions: 1.0 g AG-MP1 in a 10 cm × 1 cm ID support; flow rate ≈ 2 mL/min.

The γ-spectra of the irradiated target and subsequent purification steps are shown in Fig. 3. The following isotopes were detected: ^91^Sr, ^91,91m^Y, ^95,97^Nb, ^95,97^Zr, ^99^Mo, ^99m^Tc ^103^Ru, ^105^Rh, ^132^Te, ^131,132,133^I, ^140^Ba, ^140^La, ^141^Ce, ^147^Nd, ^149^Pm, ^237^U, and ^239^Np. All three irradiated targets were processed similarly with respect to UREX and MoLLE. However, two different approaches were adopted during the processing of the concentration columns (Figs. 4 and 5). The results are discussed below.

### UREX

The UREX decontamination step removed > 99% of the bulk uranium using excess volume 30% TBP. Varying amounts of Np, I, Nb, and Zr were observed in the loaded organic phases. In some cases, more than 50% of the Np and I were co-extracted which is to be expected given the multi-valence equilibrium of Np(IV, V, VI) and the organic solubility of elemental I_2_. The Mo remained in the raffinate along with alkaline earths, lanthanides, Np, Y, Te, I, Nb, Zr, Rh, and Ru.

### MoLLE

The UREX raffinate (containing most fission products) was then contacted with HDEHP in *n*-dodecane. This step primarily extracted Mo, Nb, Zr, Np, and small amounts of Te and I. The elements that remained in the aqueous phase were Sr, Ba, lanthanides, Y, Ru, and Rh. The AHA contact on the loaded HDEHP solvent quantitatively stripped Mo along with a significant amount of Nb after two contacts. Trace amounts of Zr, Te, and I were also observed. Table 1 lists selected isotope percentages found in the MoLLE process steps.

**Table 1:** Total activity percent for selected fission products and Np within MoLLE derived from the irradiated uranyl sulfate target (LEU).

Isotope	MoLLE Feed	HDEHP Contact 1	HDEHP Contact 2	AHA Contact 1	AHA Contact 2
^141^Ce	99	Trace^a^	Trace	Trace	Trace
^132^Te	105^b^	Trace	Trace	Trace	Trace
^239^Np	53	13	7	Trace	Trace
^131^I	51	Trace	Trace	Trace	Trace
^103^Ru	105	–	–	–	–
^140^Ba	103	–	–	–	–
^95^Zr	79	83	4	Trace	Trace
^95^Nb	87	93	5	7	12
^99^Mo	98	112	6	104	–

^a^< 1% or below quantification limits.

^b^Values > 100% reflect γ-peak overlap or changes in attenuation and density across samples.

The MoLLE extraction step provides a unique opportunity to decontaminate Mo from the selected fission products. Under mild to medium nitric acid concentrations—like those observed in UREX raffinates—HDEHP acts as a solvating extractant that typically targets highly charged IV, V, and VI valence cations^12^. Molybdenum is extracted as the solvated molybdenyl nitrate complex MoO_2_(NO_3_)_2_^8^. Consequently, HDEHP will also remove Nb(V), Zr(IV), as well as Np(IV,VI). Any residual U(VI) will also partition into the organic phase. The I, II, III, and VII valence ions do not extract including Ru and Rh which are known to exhibit complicated speciation in acid or alkaline media^13^. Ruthenium, ^103^Ru in particular, is a common impurity that must meet strict purity specifications^5^.

Once extracted into HDEHP, a slightly acidic solution of AHA can be used to recover the Mo. It was found that concentrations of AHA less than 0.3 M resulted in slow phase separation as well as an insoluble, white component at the interface that was difficult to remove.The quantity of Mo stripped from the organic phase was also insufficient. Increasing the concentration of AHA to 0.5 M improved the phase separation along with > 99% Mo back-extraction after one contact. We are also exploring the effects of pH on the AHA recovery step to improve phase separation.

### Concentration column

A final purification step was necessary to both purify and concentrate the Mo derived from MoLLE. An anion-exchange platform was selected given the high retention of Mo across a number of different acid or alkaline environments. The AHA solution used to strip Mo resulted in a feed stream also comprised of significant quantities of Nb and trace amounts of Np, Zr, Te, and I (Table 1). The solution was contacted with concentrated NH_4_OH to promote MoO_4_^2–^ formation and loaded onto the column by gravity flow. The elution profiles are shown in Fig. 4. The Zr, Np, and Te activities were easily removed with 0.1 M NaOH. Increasing the alkalinity to 1.0 M NaOH removed the remaining Te. When the concentration of NaOH was increased to 2.0 M, more Nb was recovered but with significant amounts of tailing. It was anticipated that this step would remove Nb given its ability to separate V from Mo^14^. Finally, using a 1:1 mixture of 1.0 M NaOH with 1.0 M NaCl, Mo was stripped from the column with residual Nb. The spent column was analyzed and found to contain 23% of the Nb as well as 100% of the I.

Niobium presented a significant decontamination challenge. Not only is Nb a major fission yield and present in large quantities, but in-growth from ^95^Zr is feasible in the context of long-term HDEHP solvent recycling. It should also be noted that approximately 15–20% of the total activity that entered the concentration column corresponded to Nb. Nearly 80% of the total activity came from ^99^Mo; isotopes of Zr, Np, I, and Te fulfilled the balance. In an attempt to decontaminate Nb in a single elution step, we adopted an approach that utilized the sorption capabilities of anionic chloride complexes in acid. It is known that anion-exchange resins exhibit a high affinity for Mo in 3–8 M HCl on account of the multiple anionic chloride complexes of molybdenyl, whereas Nb exhibits a minimum in partitioning between 4 and 8 M HCl^15,16^. The separation bands of Nb and Mo can be resolved even further by the addition of oxalic acid or hydrogen peroxide^17^. Leveraging this chemistry, we sought the complete removal of Nb using HCl and oxalic acid.

The MoLLE exit stream derived from the uranyl sulfate targets was loaded onto an anion exchange resin and treated using mixtures of alkali and acid. The elution profiles and results are shown in Fig. 5. As previously observed, the alkali wash steps removed Np, Zr, and a small percentage of Nb and Te. In one instance, traces of ^141^Ce (145 keV) were observed in the hydroxide washes which was unexpected given the efficient decontamination from lanthanides in MoLLE. However, this activity was only observed while processing the uranyl sulfate target and not U_3_O_8_. Tetravalent cerium is rather stable in sulfuric acid solutions^18^ and it is not unreasonable to propose that Ce(IV) was extracted into HDEHP during MoLLE. This would also be consistent with Th(IV) extraction data using HDEHP from mild nitric acid^19^. Regardless, the ^141^Ce activity was removed from the column with NaOH. Replacing the 2 M NaOH wash step (Fig. 4) with a mixture of 5 M HCl and 0.2 M oxalic acid showed promising removal of Nb, albeit a significant amount of tailing. Over 40 mL of 5 M HCl/0.2 M oxalic acid was required to remove Nb. Small traces of iodine were observed during the processing of the uranyl sulfate and were eluted along with Nb. The eluate was converted to 4 M HCl to remove traces of oxalic acid along with more iodine activity. It should also be noted that this behavior was not consistent during any trial and that iodine fractionation may not be replicable within each batch. The HCl washes, however, were capable of removing iodine from the concentration column. Finally, the 1.0 M NaOH/1.0 M NaCl mixture stripped the remaining Mo in approximately 10 mL. The overall recovery of ^99^Mo from the irradiated target was quantified at 96%.

Throughout the elution profile shown in Fig. 5, the speciation of Mo was presumed to have converted between molybdenyl-hydroxamic acid, molybdate, molybdenyl-chloride, and back to molybdate. The efficacy of the column during these transitions was somewhat surprising given that these resins exhibit low Mo(VI) retention values between 1 and 2 M HCl^20^. More recent works have resolved the anion-exchange sorption of Mo as a function of its III, V, and VI valences indicating that Mo(VI) is strongly retained across wide concentrations of HCl^21^. The NaOH/NaCl recovery step was consistently effective in all trials.

There are a number of variables that remain to be investigated as the process is scaled up and optimized. The radiological degradation of these sorbents at high activities (> 100 Ci ^99^Mo with additional fission products) as well as loading capacity in the context of multiple product batches of ^99^Mo is important. We also note that higher volumes of Mo-AHA (< 20 mL) feed may require larger columns and potentially more wash steps than those described here as some ^99^Mo was lost to the hydroxide washes in a separate experiment. We also intend to resolve the full solvent extraction/concentration column behavior of In, Sb, Sn, Cd, As, and Br as irradiations are ramped up to higher production levels.

## Conclusion

A new separation process was developed to purify and recover high-specific activity ^99^Mo from an accelerator-irradiated U_3_O_8_ or uranyl sulfate targets. The process relies on acidic solvent extraction (UREX + MoLLE) and an anion exchange column (concentration column). The extraction steps were efficient in removing the bulk uranium and isolating ^99^Mo from the majority of fission products using mild HNO_3_ and AHA. Finally, to concentrate and decontaminate ^99^Mo from trace fission products, the anion exchange column in tandem with a variety of hydroxide and HCl eluents resulted in a purified high specific activity ^99^MoO_4_^2–^ product.
