# Publisher Correction: Recovery of high specific activity molybdenum-99 from accelerator-induced fission on low-enriched uranium for technetium-99m generators

**DOI:** 10.1038/s41598-021-96494-2

**Published:** 2021-08-20

**Authors:** M. Alex Brown, Nathan Johnson, Artem V. Gelis, Milan Stika, Anna G. Servis, Alex Bakken, Christine Krizmanich, Kristin Shannon, Peter Kozak, Amanda Barnhart, Chad Denbrock, Nicolas Luciani, Terry Grimm, Peter Tkac

**Affiliations:** 1 Chemical and Fuel Cycle Technologies, Argonne National Laboratory, 9700 S Cass Ave, Lemont, IL 60439 USA; 2 Niowave, Inc., 1012 N Walnut St., Lansing, MI 48906 USA; 3 Radiochemistry Department, University of Nevada Las Vegas, Las Vegas, NV 89154 USA

Correction to: *Scientific Reports* 10.1038/s41598-021-92704-z, published online 24 June 2021 

The original version of this Article contained an error in the copyright line.

“© The Author(s) 2021”

now reads:

“© UChicago Argonne, LLC, Operator of Argonne National Laboratory, Niowave Inc, and the Author 2021”

The original Article has been corrected.

